# Understanding Camouflaging as a Response to Autism-Related Stigma: A Social Identity Theory Approach

**DOI:** 10.1007/s10803-021-04987-w

**Published:** 2021-03-31

**Authors:** Ella Perry, William Mandy, Laura Hull, Eilidh Cage

**Affiliations:** 1 Department of Psychology, Royal Holloway, University of London, Egham, UK; 2 Research Department of Clinical, Educational and Health Psychology, University College London, London, UK; 3 Division of Psychology, Faculty of Natural Sciences, University of Stirling, Stirling, Scotland

**Keywords:** Camouflaging, Stigma, Autistic identity, Psychological wellbeing, Social Identity Theory

## Abstract

Camouflaging refers to strategies used by autistic people to mask or hide social difficulties. The current study draws on Social Identity Theory to examine the relationship between camouflaging and autism-related stigma, testing the hypothesis that camouflaging represents an individualistic strategy in response to stigma. Two hundred and twenty-three autistic adults completed an online survey measuring perceived autism-related stigma, individualistic and collective strategies, camouflaging and mental wellbeing. Results indicated that higher camouflaging was positively associated with autism-related stigma and both individualistic and collective strategy use. Autism-related stigma was associated with lower wellbeing however this relationship was not mediated by camouflaging. These findings demonstrate how stigma contributes to camouflaging and highlight the complexities of navigating autistic identity while still camouflaging.

## Introduction

Autistic people show differences in social communication and interaction, focused interests and sensitivity to sensory stimulation (American Psychiatric Association, 2013). Although autism is identifiable from infancy, diagnosis occurs across the lifespan, with many autistic people not diagnosed until adulthood. Diagnosis in adulthood is potentially related to camouflaging of autistic characteristics (Hull et al., 2017). Camouflaging refers to strategies that mask social difficulties and enable “passing” as though non-autistic in social situations (Hull et al., 2017; Livingston et al., 2020). It can include the use of techniques to appear socially competent, such as rehearsing facial expressions, eye contact and social scripts (Bargiela et al., 2016).

Understanding camouflaging is relevant to the mental health of autistic people. Qualitative research has shown autistic people discuss camouflaging in relation to experiencing greater mental and physical exhaustion (Bargiela et al., 2016; Hull et al., 2017; Tierney et al., 2016). Quantitative studies have demonstrated associations between self-reported camouflaging and depression, anxiety, stress, social anxiety, suicidality and poor well-being (Beck et al., 2020; Cage & Troxell-Whitman, 2019; Cage et al., 2018; Cassidy et al., 2018; Hull et al., 2019, 2021). Given the high prevalence of mental health difficulties in autistic people (Lai et al., 2019), understanding behaviours that negatively impact on psychological wellbeing, such as camouflaging, is imperative.

Researchers have discussed two broad reasons for camouflaging—first, to fit in to a non-autistic world and second, to maintain relationships. For example, Hull et al. (2017) found that autistic people camouflaged due to desire to assimilate, connect with others and avoid exclusion or discrimination. Late-diagnosed autistic women associate camouflaging with attempting to fit in, describing the effort involved as exhausting and confusing for identity (Bargiela et al., 2016). In interviews with female autistic adolescents, participants described a desire to make friends and gain acceptance following rejection (Tierney et al., 2016). Cage and Troxell-Whitman (2019) found that autistic adults reported camouflaging to pass in the non-autistic world, avoid bullying and manage others’ impressions of them.

One underlying explanation for existing findings is that camouflaging represents a response to autism-related stigma (Cage & Troxell-Whitman, 2019; Pearson & Rose, 2021). Long-term management of stigma depletes psychological resources, leading to difficulties regulating emotions, often cited as the core of mental health difficulties (Hatzenbuehler et al., 2013). Stigma can be defined as the social discrediting of attributes which causes individuals to feel unacceptable or ‘othered’ (Goffman, 1990). For autistic individuals, this might be the discreditation of autistic behaviours, such as self-stimulating (stimming) behaviours (Kapp et al., 2019), differences in social presentation (Sasson et al., 2017), or discrimination against the label of “autism” (Brosnan & Mills, 2016). As noted, key motivations for camouflaging appear to centre around fitting in, gaining acceptance and avoiding exclusion (Cage & Troxell-Whitman, 2019; Cage et al., 2018; Hull et al., 2017), aligning with the notion of managing a stigmatised identity via ‘passing’ or ‘covering’ (Goffman, 1990; Pearson & Rose, 2021).

Autistic people can be understood as an identity-based minority group affected by stigmatised social status (Botha & Frost, 2020) and autistic people often report experiencing stigma (Botha et al., 2020; Shtayermman, 2009). Research with non-autistic adults demonstrates stigma, for example, in rapid negative first impressions formed by non-autistic observers (Sasson et al., 2017) and the dehumanisation of autistic people (Cage et al., 2019). Further, depictions of autism within media, legislation, research and autism charities may promote stigmatisation (Holton et al., 2014; Nicolaidis, 2012). As such, autistic individuals are at risk of experiencing stigma (Botha & Frost, 2020) but research is needed to investigate how stigma relates to camouflaging.

One way of examining these relationships is via a Social Identity Theory (SIT; Tajfel & Turner, 2004) framework, which proposes that when a group is stigmatized, group members seek to regain a positive identity through individualistic and collective strategies. Individualistic strategies involve dissociating from one’s in-group (e.g., the autistic community) and attempting to “pass” into a higher status out-group (e.g., non-autistic communities). In contrast, collective strategies aim to benefit in-group status by positively re-defining the in-group compared to the out-group. Examples of collective strategies include joining online social networks, support groups or autism rights organisations. Camouflaging may involve dissociating from the autistic in-group to “pass” as non-autistic, thus potentially representing an individualistic strategy in response to stigma.

Considering camouflaging through this framework presents several hypotheses. If camouflaging is an individualistic strategy, it indicates that perceived stigma motivates camouflaging [since the strategies are proposed in response to stigma, as outlined above (see also Nario-Redmond et al., 2013)]. This notion is supported by Botha and Frost’s (2020) finding that autistic participants who more frequently concealed their autistic traits (e.g. not disclosing autistic status to others) also reported more internalised stigma (acceptance and application of negative stereotypes and stigma around autism to one’s self) and experiences of discrimination. They used a 5-item measure to examine ‘concealment’ (conceptually similar to camouflaging) but did not look at the relationship with perceived autism stigma (i.e., how much autistic people think other people stigmatise autism), only internalised stigma. As such, the relationship between perceived stigma and camouflaging requires further quantitative investigation to support, extend and complement pre-existing research—and to the best of our knowledge, this relationship has not yet been investigated quantitatively using the discussed SIT framework of individualistic and collective strategies.

The proposed framework also suggests hypotheses around psychological wellbeing. We would posit direct relationships between stigma and wellbeing, following a minority model approach, whereby autistic people have a stigmatised minority identity and are subject to greater stress due to stigmatisation and discrimination (Botha & Frost, 2020). Given our above discussion regarding camouflaging as a response to stigma, it is worth examining camouflaging as a mediator in the relationship between stigma and wellbeing. For example, Hull et al. (2017) found that autistic people described feeling they had betrayed the autistic community by camouflaging, and camouflaging obstructed their relationships with other people. Camouflaging could thus validate stigma and undermine connections to one’s in-group, potentially eliciting shame and reducing access to in-group support—something which has been highlighted as a potential buffer against mental health difficulties for autistic adults (Cooper et al., 2017). Prior research has also highlighted the relationships between camouflaging and wellbeing (e.g., Cage & Troxell-Whitman, 2019; Cassidy et al., 2018; Hull et al., 2019, 2021). Thus, we aimed to examine how camouflaging could potentially mediate any relationship between stigma and wellbeing.

Therefore, the current study aimed to investigate whether camouflaging could be understood as an individualistic strategy in response to autism-related stigma. We hypothesised, based on the above literature, that: (1) stigma positively relates to camouflaging, (2) camouflaging mediates the relationship between stigma and wellbeing, (3) individualistic strategy use positively relates to camouflaging. We also predicted a relationship between camouflaging and collective strategy use (non-directional hypothesis).

## Methods

### Participants

Two hundred and twenty-three participants took part in the study. One hundred and thirty (58.3%) identified as female, 53 as male (23.8%), 39 identified as non-binary or used other gender terminology (17.5%) and one participant preferred not to say (0.4%). Participants’ ages ranged from 18 to 65 years (M = 34.19, SD = 11.00), and age at diagnosis ranged from 2 to 63 years (M = 28.67, SD = 13.31). Participant characteristics are presented in Table 1, indicating that the sample was mostly White, lived in the United Kingdom and were university educated.

**Table 1:** Participant characteristics including education, ethnicity, current country and preferred terminology

	%
Education	
None	3.6
High school	11.7
College/sixth form	22.4
Trade/vocational	4.9
Undergraduate degree	28.7
Masters degree	17.0
Doctorate	6.7
Other	2.2
Preferred not to say	2.7
Ethnicity	
White	92.8
Mixed	3.1
Other	1.3
Black	0.4
Preferred not to say	2.2
Current Country	
United Kingdom	65.9
North America (United States or Canada)	19.7
Other European country	11.2
Australia or New Zealand	2.2
United Arab Emirates	0.4
Preferred not to say	0.4
Preferred terminology	
Autistic person	57.8
Person with autism	12.1
No preference	26.5
Other	3.6

One hundred and sixteen participants self-reported a diagnosis of ‘Asperger’s Syndrome’, 105 reported the diagnosis of ‘Autism’ or ‘Autism Spectrum Condition/Disorder’, and two reported ‘Pervasive Developmental Disorder—Not Otherwise Specified’. Presence of autistic characteristics was confirmed using the Ritvo Autism and Asperger Diagnostic Scale (RAADS-14; Eriksson et al., 2013). All participants scored above the cut-off score of 14 (range 14–42, M = 34.14, SD = 6.26).

We recruited participants via online and offline communities through snowballing methods (e.g., adverts posted on the researchers’ social media accounts (Twitter, Facebook, Instagram, Reddit), emails to UK-based autism community groups (e.g., for support and advocacy), charities (who shared the advert with relevant people) and word-of-mouth via personal contacts) between November 2018 and January 2019. All participants gave informed consent before participating and ethical approval was granted by the Research Ethics Committee at Royal Holloway, University of London.

### Materials and Procedure

Participants completed an online survey using the Qualtrics survey platform, completing the measures described in the order below. Following participatory research guidelines (Fletcher-Watson et al., 2019), the survey was developed in consultation with an autistic person who completed a semi-structured interview regarding the relevance of the study to the autistic community, the readability and cultural sensitivity of the survey and estimated completion time. Due to funding limitations, the study regrettably lacked deeper autistic involvement.

#### Terminology Preference

Participants could select their preferred terminology to customise the survey (e.g., ‘person with autism’ or ‘autistic person’). Based on participant’s selections, where relevant, they saw their preferred terms. Since most preferred identity-first (57.8%) or had no preference (26.5%), we use identity-first terminology throughout this paper.

#### Individualistic Strategy Use

Nario-Redmond et al.’s (2013) 13-item measure of individualistic strategy use was adapted (e.g. replacing ‘disabled person’ with ‘autistic person’). Items related to components denying or minimalising the importance of autism (e.g., ‘I don’t think of myself as an autistic person’), striving to “overcome” autism (‘I do not need to be “cured” of autism’) and concealing being autistic (‘I try to hide autistic behaviours whenever I can’). Participants rated each item using a 7-point Likert scale [‘strongly disagree’ (1) to ‘strongly agree’ (7)]. Scores could range between 13 and 91—higher scores indicated greater use of individualistic strategies. In this sample, internal consistency was good (Cronbach’s α = 0.77).

#### Collective Strategy Use

Nario-Redmond et al.’s (2013) 13-item measure of collective strategy use was adapted as above. Items related to expression of community or community pride (‘Autism culture is alive and well’), valuing experience (‘Autism enriches my life’) and support for social change (‘I am an autism rights activist’). Participants rated items using the 7-point scale as above. Higher scores indicated greater use of collective strategies. Internal consistency was very good (α = 0.89).

#### Stigma Consciousness Scale

The Stigma Consciousness Scale (Link & Phelan, 2014) assesses awareness of stigmatised status. The original scale related to mental illness, thus was adapted, for example ‘People knowing that I am autistic does not influence how they act towards me’. Participants rated five items on a 4-point scale [‘strongly agree’ (0) to ‘strongly disagree’ (3)]. Scores could range between 0 and 15. Higher scores indicated greater awareness of stigmatisation. Internal consistency was questionable (α = 0.65).

#### Camouflaging Autistic Traits Questionnaire

The Camouflaging Autistic Traits Questionnaire (CAT-Q; Hull et al., 2019) is a 25-item measure of self-reported camouflaging. Example items include ‘In my own social interactions, I use behaviours that I have learned from watching other people interacting’. Participants rated each item on the same 7-point Likert scale as above. Scores could range between 25 and 175—higher scores indicated greater camouflaging. Internal consistency was excellent (α = 0.90).

#### Warwick–Edinburgh Mental Wellbeing Scale

The Warwick–Edinburgh Mental Wellbeing Scale [WEMWBS (Tennant et al., 2007)], is a 14-item measure of psychological wellbeing. Example items include ‘I’ve been feeling relaxed’ and ‘I’ve been interested in new things’. Participants rated items on a 5-point Likert scale [‘none of the time’ (1) to ‘all of the time’ (5)]. Scores could range from 14 to 70, and higher scores indicated more positive mental wellbeing. Internal consistency was excellent (α = 0.92).

#### Ritvo Autism and Asperger Diagnostic Scale

The RAADS-14 (Eriksson et al., 2013) is a 14-item screening tool for autistic characteristics. Items relate to experiences of social interactions, sensory stimulation and routine, for example, ‘I focus on details rather than the overall idea’. Participants rated items on a 4-point Likert scale [‘never true’ (0), ‘true only when I was younger than 16′ (1), ‘true only now’ (2) and ‘true now and when I was young’ (3)]. Scores could range from 0 to 42 with higher scores indicating greater autistic traits. Internal consistency was acceptable (α = 0.72).

#### Demographic Questions

Finally, participants reported their age, age at diagnosis, official diagnosis, gender, ethnicity, and level of education.

### Design and Data Analysis

This study had a cross-sectional correlational design. Statistical analyses were conducted in SPSS version 25 with the PROCESS add-on version 3.3 (Hayes, 2012) for mediation analysis. Two dummy variables were created for gender; ‘female versus male’ and ‘female versus non-binary’, with female as the reference category due to this being the largest group in the study. A priori power analysis (with predicted power at 0.80; Cicchetti et al., 2011) indicated that a sample size of 98 participants would be suitable. For all analyses, assumptions were met: for regression, there was no multicollinearity (VIF values 1.02–1.55), data were normally distributed and homoscedastic, variable relationships were linear and independent and there were no influential outliers. For the mediation analysis, assumptions of data linearity, normality and independence were met. We considered *p* values between 0.05 and 0.005 as suggestive and *p* < 0.005 as our significance threshold (Ioannidis, 2018). We also report confidence intervals and effect sizes as appropriate.

We assessed hypothesis one (stigma and camouflaging) using multiple regression with camouflaging as the dependent variable, and stigma and demographic variables (age, age at diagnosis, gender and autistic traits) as independent variables. These variables were controlled for since previous research has found all four relate to camouflaging (e.g. Hull et al., 2019), and there were indicative correlations in our study (Table 3). Thus, our model tests whether, after controlling for these covariates, our hypothesis is still met. We tested hypothesis two (camouflaging mediates between stigma and wellbeing) by using mediation analysis, with wellbeing as the dependent variable, stigma the independent variable and camouflaging the mediator. We investigated hypothesis three (strategy use and camouflaging) using multiple regression with camouflaging as the dependent variable. Demographic variables (as above), individualistic strategy and collective strategy use were entered as independent variables.

## Results

Means and standard deviations for each of the independent variables are presented in Table 2 and correlations between variables are shown in Table 3.

**Table 2:** Means, standard deviations and range of scores for independent variables in the study

	Mean (SD)	Range	Skewness*	Kurtosis*
Collective strategy use	63.96 (13.40)	13–91	− 0.57	0.40
Individualistic strategy use	44.74 (10.77)	16–76	0.23	0.011
Stigma	10.61 (2.48)	3–15	− 0.22	− 0.31
Camouflaging	126.65 (21.79)	61–165	− 0.61	0.13
Wellbeing	39.07 (9.56)	14–69	0.13	0.24

*Values between − 2 and + 2 considered acceptable in relation to normal distribution (Byrne, 2010; Hair et al., 2010)

**Table 3:** Correlations between variables included in the study

	Age	Age at diagnosis	Female versus male	Female versus non binary	Collective strategies	Individualistic strategies	Stigma	CATQ	WEMWBS
Age at diagnosis	0.87**								
Female versus male	0.10	0.019							
Female versus non-binary	− 0.064	− 0.070	− 0.26***						
Collective strategies	− 0.009	0.018	− 0.18**	0.12					
Individualistic strategies	− 0.072	− 0.077	− 0.018	− 0.11	− 0.52**				
Stigma	0.057	0.11	− 0.018	0.21***	0.12	− 0.29***			
CATQ	0.053	0.17**	− 0.15*	− 0.020	0.14*	0.079	0.30***		
WEMWBS	0.012	− 0.067	− 0.016	− 0.014	0.25**	0.025	− 0.21**	− 0.16*	
RAADS	0.043	0.17*	− 0.061	− 0.040	0.076	− 0.28***	0.24***	0.19**	− 0.26***

***p < 0.001, **p < 0.01, *p < 0.05, two-tailed

### Stigma and Camouflaging

**Fig. 1:**
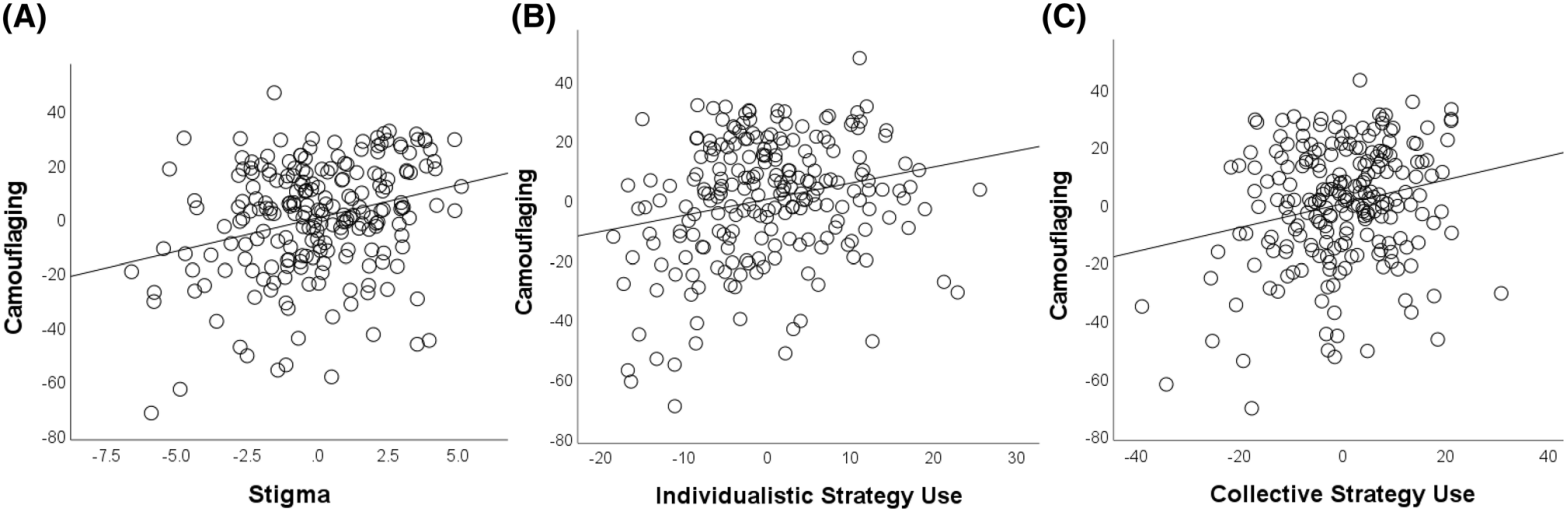
Partial regression plots showing: **a** The relationship between stigma and camouflaging total scores. **b** The relationship between individualistic strategy and camouflaging total scores. **c** The relationship between collective strategy and camouflaging total scores

The model was significant (*F*(6, 218) = 7.12, *p* < 0.001, *f*^2^ = 0.17) and accounted for 14.4% of the variation in camouflaging (Table 4). Stigma significantly predicted camouflaging, such that with increasing stigma scores, camouflaging scores increased (Fig. 1). Additionally, age, age at diagnosis and gender were suggestive predictors of camouflaging, such that older age related to less camouflaging and older age of diagnosis and not being male predicted more camouflaging.

**Table 4:** Regression model testing the relationship between stigma and camouflaging

Predictor	B	B CI	SE B	β	*p*	*f*^2^
Age	− 0.53	[− 1.04, − 0.022]	0.26	− 0.27	0.041	0.017
Age at diagnosis	0.59	[0.16, 1.02]	0.22	0.36	0.007	0.029
Female versus male	− 7.62	[− 14.25, − 0.99]	3.36	− 0.15	0.024	0.019
Female versus non-binary	− 6.47	[− 14.05, 1.12]	3.85	− 0.11	0.095	0.009
Autistic characteristics	0.25	[− 21, 0.71]	0.23	0.072	0.28	0.005
Stigma	2.42	[1.26, 3.57]	0.58	0.28	< 0.001	0.069

B unstandardised beta coefficient, B CI confidence intervals at 95% lower and upper bounds, SE B standard error, β standardised beta coefficient, *f*^2^ individual predictor effect size (effect size 0.02 considered small, 0.15 medium)

### Camouflaging, Stigma and Wellbeing

**Fig. 2:**
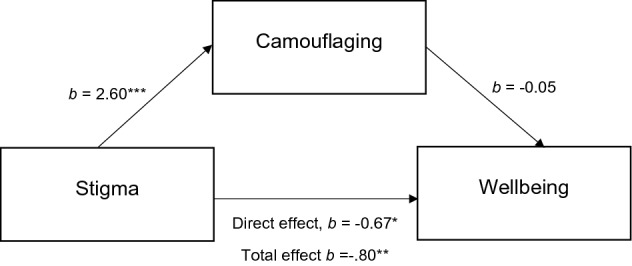
Mediation model examining the relationships between stigma, camouflaging and wellbeing. *p = 0.012, **p = 0.002, ***p < 0.001

The total effect (sum of all effects) was significant (*b* = − 0.80, *t*(223) = − 3.16, *p* = 0.002). The path between stigma and camouflaging was significant (*b* = 2.60, t(223) = 4.59, *p* < 0.001) but the path between camouflaging and wellbeing was not (*b* = − 0.05, t(223) = − 1.65, *p* = 0.10). The direct effect (unmediated effect of stigma on wellbeing, with camouflaging held constant) was suggestively significant (*b* = − 0.67, t(223) = − 2.55, *p* = 0.012). The indirect effect was not significant (a*b = − 0.13), with confidence intervals including zero [− 0.34–0.032] (Fig. 2).

### Camouflaging and Strategy Use

Overall, the model was significant, *F*(7, 218) = 5.63, *p* < 0.001, *f*^2^ = 0.15) and accounted for 12.9% of the variation in camouflaging (Table 5). Individualistic strategy use and collective strategy use were both significant predictors (Fig. 1): with increases in both strategies, camouflaging also increased. Further, age at diagnosis and autistic characteristics were significant predictors, such that with later age of diagnosis and greater autistic characteristics there was more camouflaging.

**Table 5:** Regression model examining the relationship between individualistic and collective strategy use and camouflaging

Variable	B	B CI	SE B	β	*p*	*f*^2^
Age	− 0.52	[− 1.04, − 0.001]	0.26	− 0.26	0.050	0.015
Age at diagnosis	0.63	[.20, 1.07]	0.22	0.38	0.005	0.032
Female versus male	− 3.96	[− 10.80, 2.89]	3.47	− 0.078	0.26	0.005
Female versus non-binary	− 2.13	[− 9.71, 5.45]	3.85	− 0.037	0.58	0.001
Autistic characteristics	0.72	[0.24, 1.20]	0.25	0.20	0.004	0.034
Individualistic strategy use	0.54	[0.22, 0.86]	0.16	0.27	0.001	0.044
Collective strategy use	0.41	[0.16, 0.66]	0.13	0.25	0.001	0.042

## Discussion

The present study examined the relationships between stigma, individualistic and collective strategy use and camouflaging in a sample of autistic adults. In summary, we found that higher perceived autism stigma predicted higher levels of self-reported camouflaging, and higher individualistic and collective strategy use also predicted more camouflaging. Autism-related stigma had a negative relationship with mental wellbeing (including when camouflaging was controlled for), however there was no mediation effect, suggesting that stigma does not influence wellbeing via increased camouflaging, and our initial hypothesis was not supported here.

These findings quantitatively show the relationship between camouflaging and experiences of stigma. Our findings fit with qualitative data from autistic people on the motivations for camouflaging (e.g. Bargiela et al., 2016; Cage & Troxell-Whitman, 2019; Hull et al., 2017), and their reports of experiencing more bullying and harassment when not camouflaging (Cage & Troxell-Whitman, 2019). Our findings also support research examining autistic people’s experiences through a minority model (Botha & Frost, 2020). Here, it is argued that stigmatised minority groups experience greater social stress, contributing to greater physical and mental health difficulties (Schwartz & Meyer, 2010). Botha and Frost (2020) found that minority stressors such as behavioural concealment (similar to camouflaging but also related to aspects of disclosure) related to internalised stigma. Although our approach looked at camouflaging through Social Identity Theory rather than a minority stress model, together these findings show the utility of applying social theories to our understanding of camouflaging (Pearson & Rose, 2021). Our findings reinforce the link between perceptions of stigma and camouflaging: Camouflaging manifests in response to being ‘othered’ and feeling pressurised to conform to non-autistic social conventions to avoid stigmatisation (Hull et al., 2017; Pearson & Rose, 2021).

In terms of strategy use, greater individualistic strategy use predicted more camouflaging. This finding supports qualitative research where accounts of camouflaging bear similarities to descriptions of individualistic strategies (e.g. referring to camouflaging as “pretending to be normal” or “passing”; Cage & Troxell-Whitman, 2019; Hull et al., 2017). However, greater collective strategy use also predicted more camouflaging, which undermines camouflaging solely representing an individualistic strategy. Individualistic and collective strategies were negatively correlated, supporting the assumption that these are contrasting strategies (i.e. rejecting versus embracing the stigmatised in-group; Tajfel & Turner, 2004).

Accordingly, our findings show how camouflaging co-occurs with advocating for the autistic community and strongly identifying as autistic. Autistic people’s qualitative accounts of camouflaging have described pride in being autistic, but continuing to camouflage specific behaviours which may be considered socially unacceptable (Hull et al., 2017) and research has shown how disclosure may mediate the relationship between positive autistic identity and reduced camouflaging (Cage & Troxell-Whitman, 2020). Camouflaging persists so long as autism is stigmatised: those using collective strategies may have heightened awareness of stigma and non-autistic people’s lack of tolerance and expectations around ‘normalcy’ (see Goffman, 1990; Milton, 2013). For example, social interaction partners form negative judgements if someone violates social norms, such as making little eye contact or not using conventional means to show social interest (Sasson et al., 2017). Thus, stigmatised behaviours and identities are camouflaged, as autistic people are forced to weigh up personal and social costs of camouflaging against potential gains, such as protection against discrimination (Cage & Troxell-Whitman, 2019).

Counter to our hypothesis, stigma was directly negatively related to wellbeing (controlling for camouflaging), although this was only significant at a suggestive *p*-value threshold—thus, caution is warranted. We did not find a mediation effect with camouflaging playing any role in the relationship between stigma and wellbeing. Interestingly, within our mediation model camouflaging did not significantly predict wellbeing, counter to pre-existing research (e.g. Cage & Troxell-Whitman, 2019; Cassidy et al., 2018; Hull et al., 2019). Accordingly, our findings highlight that there is potentially more of a direct relationship between stigma and wellbeing, which camouflaging does not mediate. This finding fits with the minority stress model described previously (Botha & Frost, 2020). Since stigma and camouflaging were related in all analyses, stigma plays some role in camouflaging, but there is no mediating role onto wellbeing. Alternatively, camouflaging may affect wellbeing differently for different individuals, and any mediation effect is cancelled out and non-significant. For example, the mediation analysis did not account for gender, where past research has noted potential gender differences in terms of relationships between camouflaging and wellbeing outcomes (Lai et al., 2017). Further, we measured the general concept of mental wellbeing, whereas other studies have focused on specific aspects of mental health such as depression, anxiety and social anxiety, and shown differences in the relationships between these different conditions and camouflaging (e.g. Hull et al., 2021). Given the self-reported impacts of camouflaging on mental health (e.g. Bargiela et al., 2016; Hull et al., 2017), investigations into this topic are still worthwhile. Our findings do support calls to target stigma and conditions within the external environment which appear to necessitate camouflaging, rather than trying to change the autistic individual (Mandy, 2019).

In our analyses, several demographic variables also had relationships with camouflaging. Later diagnosis was a predictor of greater camouflaging, supporting the proposition that camouflaging may reduce the likelihood of obtaining a timely autism diagnosis (Hull et al., 2017; Lai et al., 2017), although this relationship could be bidirectional with those late-diagnosed perhaps being more likely to persistently camouflage. Greater autistic characteristics predicted increases in camouflaging in the model with strategies but was not significant in the model with stigma. Although autistic characteristics significantly correlated with both stigma and individualistic strategies, the regression findings suggest the relationship between autistic characteristics and camouflaging may result from stigma, such that when stigma is included in the model, no direct relationship between autistic characteristics and camouflaging is observed. Botha and Frost (2020) speculated that confirmation of the label ‘autism’ may link to more experiences of and internalisation of stigma. The present findings could indicate that autistic characteristics relate to camouflaging via their relationship with stigma, through the extent to which they increase one’s awareness of stigma and feeling that more camouflaging is necessary to hide autistic characteristics. For gender, we found only suggestive to null findings, which overall suggest little to no relationship between gender and camouflaging in our statistical models. This support previous work which has also not found a relationship between camouflaging and gender (Cage & Troxell-Whitman, 2019; Cassidy et al., 2020) and calls to use caution when discussing camouflaging in relation to gender (Pearson & Rose, 2021).

### Implications and Future Research

The current study supports the need for education and stigma interventions for the non-autistic population. Stigma reduction programs aimed at non-autistic adolescents and university students are found to reduce stigma and increase knowledge of autism (Gillespie-Lynch et al., 2015; Obeid et al., 2015; Ranson & Byrne, 2014; Staniland & Byrne, 2013). Attention should also be paid to the role that ideological orientations of organisations may have in stigmatising autism and necessitating camouflaging (Bottema-Beutel et al., 2018). For example, Gillespie-Lynch et al. (2017) found less interest in “normalising” autistic people was associated with lower stigma towards autism. Bottema-Beutel et al. (2018) recommend social skills interventions shift focus from enforcing normative expectations to sharing information about non-autistic social interactions and encouraging autistic people to appraise these social arrangements rather than conform to them (Bottema-Beutel et al., 2018).

The current study also has implications for clinicians and practitioners working with autistic people. As diagnostic tools do not currently assess for the presence of camouflaging (e.g. Lord et al., 2000; Mandy et al., 2018), and camouflaging strategies may be difficult for clinicians to observe, clinical assessments may benefit from the inclusion of questions about camouflaging, such as the CAT-Q (Hull et al., 2019). Clinicians should also be aware of autistic people’s experiences of stigma: repeated stigma experiences are recognised as a form of trauma (Sweeney et al., 2016). Similarly, therapists should tactfully talk to autistic clients about camouflaging, being mindful that for some autistic people talking about camouflaging may feel like being “outed” and evoke feelings of shame (Hull et al., 2017).

### Limitations

The findings of the study are not generalisable—participants were predominantly White, female and university educated. The findings do not reflect the experiences of different groups of autistic people, particularly those with additional support needs (Pellicano et al., 2014; Russell et al., 2019). Additionally, the lack of Black, Asian or ethnic minority participants is concerning. More effort is needed to reach communities often neglected in research to consider how camouflaging may relate to the intersection of different identities, especially as those with multiple minority identities may experience heightened stigma (Balsam et al., 2011; Budge et al., 2016). Further, the measures used in this study were developed primarily with White people, introducing further bias. Jones and Mandell (2020) outline how enhancing opportunities for Black autism researchers may lead to increased ethnic diversity within autism research. However, a strength of the current study is the large proportion of female and non-binary people, who have not been well represented previously (Pellicano et al., 2014). Nonetheless, the sample is limited in recruiting via social media and autistic community groups where stronger autistic identification may be found.

The findings are also limited by the cross-sectional, correlational nature of the study and relying on self-report via an online survey. Causation and directionality cannot be inferred, and effect sizes were small throughout. Longitudinal research is required to examine the relationships between stigma, camouflaging and wellbeing over time. Further, identity, strategy use and camouflaging likely fluctuate and may be context dependent (Brune & Wilson, 2013; McDonald, 2017). Such variability is not captured at a single time point, therefore future research should investigate how camouflaging and its relationship to identity may vary across contexts (Cage & Troxell-Whitman, 2019, 2020). Additionally, we were not able to independently verify self-reported diagnoses. While online surveys have limitations, they enable a large sample of autistic people to accessibly participate. Further qualitative work examining our findings would be beneficial to validate our interpretations. Finally, there was questionable reliability of the stigma measure and we used adapted measures from the disability literature, thus it may be worthwhile developing autism-specific measures of stigma and strategies with autistic people to enhance construct validity.

Finally, it remains unclear whether camouflaging occurs in response to a stigmatised identity or stigmatised behaviours. Researchers have found that non-autistic people’s negative judgements of autistic people were improved when diagnostic labels were provided, and more positive judgements are associated with more autism knowledge (Sasson & Morrison, 2019). These findings suggest stigma may be more attached to behaviours associated with being autistic, rather than the label or identity, supporting previous research (Butler & Gillis, 2011). Accordingly, this may explain why camouflaging is also noted in non-autistic people who report higher autistic traits (Livingston et al., 2020). The measure of stigma used in the current study looked at awareness of stigma and does not tell us whether perceived stigma was based on discreditation of identity or behaviours.

## Conclusion

The present study utilised a SIT framework to quantitatively examine the relationships between camouflaging, stigma, individualistic and collective strategy use. While individualistic strategies (e.g., distancing oneself from an in group) did predict camouflaging, collective strategies (e.g., self-advocacy and community pride) also predicted camouflaging; and stigma was consistently related to camouflaging. These findings highlight the internal conflicts related to camouflaging and demonstrate how experiences of stigma should be considered when attempting to explain and understand camouflaging.
